# The Effect of Probiotic Bacteria on Composition and Metabolite Production of Faecal Microbiota Using In Vitro Batch Cultures

**DOI:** 10.3390/nu15112563

**Published:** 2023-05-30

**Authors:** Jessica Eastwood, Saskia van Hemert, Carlos Poveda, Stephen Elmore, Claire Williams, Daniel Lamport, Gemma Walton

**Affiliations:** 1 School of Psychology and Clinical Language Sciences, University of Reading, Earley Gate, Reading RG6 6BZ, UK; 2 Winclove Probiotics, Hulstweg 11, 1032 LB Amsterdam, The Netherlands; 3 Department of Food and Nutritional Sciences, University of Reading, Whiteknights, Reading RG6 6AP, UK

**Keywords:** gut microbiota, probiotics, neurotransmitters, neuroactive metabolites

## Abstract

Probiotic supplements are increasingly being used to target the gut microbiome with a view to improving cognitive and psychological function via the gut-brain axis. One possible mechanism behind the effect of probiotics is through alterations to microbially-derived metabolites including short-chain fatty acids (SCFA) and neurotransmitters. However, research to date has largely been conducted in animal models or under conditions irrelevant to the human gastrointestinal tract (GIT). The aim of the current work was therefore to use anaerobic, pH controlled in vitro batch cultures to (a) assess the production of neuroactive metabolites in human faecal microbiota under conditions relevant to the human GIT, and (b) to explore how several pre-selected probiotic strains may affect bacterial composition and metabolite production. Enumeration of bacteria was assessed using fluorescence in situ hybridisation with flow cytometry, and concentrations of SCFAs and neurotransmitters were measured using gas chromatography and liquid chromatography mass spectroscopy, respectively. GABA, serotonin, tryptophan, and dopamine were successfully detected, suggesting some level of microbial derivation. The addition of *Lactococcus lactis* W58 and *Lactobacillus rhamnosus* W198 resulted in a significant increase in lactate after 8 h of fermentation, while no significant effect of probiotics on bacterial composition or neurotransmitter production was found.

## 1. Introduction

There is now a wealth of evidence to support a complex bidirectional relationship between the gut microbiota and the brain, with several microbiota-gut-brain pathways, including the vagus nerve, microbiota-derived metabolites, immune parameters, and the neuroendocrine system, being identified as key communication routes, largely through animal models [1,2]. Modulating the gut microbiota to increase the diversity and number of beneficial microbes may positively affect neural activity and behaviour through any number of these pathways. As such, the gut microbiome is more frequently being targeted for its potential to improve cognitive and psychological function.

One approach to altering the microbiota is through use of probiotic supplements. Probiotics are defined by the World Health Organisation as live microorganisms that, when administered in adequate amounts, confer a health benefit to the host [3]. The effects of various probiotic bacteria on cognitive and psychological function have now been studied in several human trials, with some promising evidence showing an improvement of cognitive function and mood, particularly in those with relevant clinical disorders such as Alzheimer’s Disease and depression [4,5,6]. However, despite the recent increase in randomised control trials, the mechanisms underlying these effects remain evasive [7].

Intervention studies in both animals and humans have reported associated increases in lumen, serum, and neural concentrations of neurotransmitters and their precursors following chronic probiotic supplementation [8,9,10,11]. As such, there is growing interest in microbiota-derived metabolites and their role in the gut brain axis. It is becoming clear that certain strains of bacteria, including those found enterically, can produce neurotransmitters [12,13]. In silico methods show predicted changes in the abundance of gut-derived neurotransmitters such as γ-aminobutyric acid (GABA) following probiotic supplementation [14]. In addition, genome-based analyses have allowed for the cataloguing of the neuroactive potential of various bacteria strains to synthesise and utilise metabolites relevant to the gut-brain axis [15,16]. This neuroactive potential has also been explored in vitro for several promising strains, with the production of GABA [17,18], dopamine [19], serotonin [19], histamine [20], norepinephrine [21], and acetylcholine [22] from bacteria of various genera, including *Lactobacillus, Bifidobacterium*, and *Lactococcus*, being reported. However, in the majority of these studies, probiotic bacteria were cultured in conditions optimised for neurotransmitter synthesis rather than in conditions typically found in the human gastrointestinal tract. While this suggests the strains are capable of producing neurotransmitters, it is less clear to what extent this may occur under physiologically relevant conditions. The presence of several neurotransmitters was recently reported in one in vitro study utilising three-stage continuous gut models to explore the impact of a pre- or probiotic intervention on metabolite production in faecal microbiota from healthy young adults under conditions reflective of anorexia nervosa [23]. Here, relatively low concentrations of GABA, serotonin, dopamine, norepinephrine, and epinephrine were detected following a restricted nutrient phase, and provision of pre- and probiotic supplements modulated metabolite synthesis to resemble that seen during a healthy control feeding phase using standard gut model media.

In addition to neurotransmitters, gut microbes produce short-chain fatty acids (SCFAs) as a result of polysaccharide fermentation [24]. SCFAs such as butyrate, acetate, and propionate regulate the expression of precursors tryptophan 5-hydroxylase and tyrosine hydroxylase, which in turn influence the synthesis of serotonin (5-HT) and biosynthesis of catecholamines dopamine, epinephrine, and norepinephrine, respectively [25]. Further to their role in neurotransmitter synthesis, SCFAs appear to be important in the production of brain-derived neurotropic factor (BDNF), blood-brain-barrier integrity, gut permeability, and regulating neuroinflammation, all of which have a significant effect on cognitive and psychological function [26]. Although largely established through animal research, the introduction of probiotics has been found to modulate the number of SCFA-producing bacteria, and subsequently increased concentrations of SCFAs have been reported in the gut lumen [27]. As such, where probiotic bacteria may not directly produce neurotransmitters under physiologically relevant conditions, production of neuroactive compounds may instead be modulated as a result of increased SCFA synthesis.

In vitro batch culture fermentation provides a means to explore the effect of probiotics on the human faecal bacterial community and to examine metabolite production under anaerobic conditions, allowing for control of nutrient availability, pH, and temperature to mimic the environment of the human colon. As such, this work employed faecal batch culture fermentation with the primary aim of assessing the production of neuroactive metabolites in human faecal microbiota under conditions relevant to the human GIT. In addition, this work aimed to explore how a selection of probiotic strains previously deemed to have high neuroactive potential [15] may affect bacterial composition and the synthesis of both SCFAs and neurotransmitters.

## 2. Methods

### 2.1. Preparation of Probiotic Strains

Six probiotic strains (*Lactobacillus rhamnosus* W198, *Lactobacillus reuteri* W192, *Bacillus subtilis* W201, *Bacillus coagulans* W64, *Propionibacterium freudenreichii* W200, and *Lactococcus lactis* W58, supplied by Winclove Probiotics) were selected for inclusion based on previous metagenomic work identifying the neuroactive potential to synthesise relevant neurotransmitters and short-chain fatty acids [15]. Prior to performing the batch cultures, calibration curves in Man Rogosa Sharpe broth (Sigma-Aldrich, Kent, UK) for *L. rhamnosus* W198, *L. reuteri* W192, *B. coagulans* W64, *Propionibacterium freudenreichii* W200, and *L. lactis* W58 and General Nutrient Broth (Sigma-Aldrich, Kent, UK) for *B. subtilis* W201 were conducted in triplicate for each strain in order to identify the correlation between optical density (OD_600nm_) (Thermo Scientific Orion AquaMate 8000 (Waltham, MA, USA)) and bacterial numbers in colony forming units (CFU).

In preparation for batch cultures, Hungate tubes containing the appropriate anaerobic broth (detailed above) were inoculated with a colony of bacteria. These were incubated overnight at 37 °C, after which cultures were measured for OD, and this was adjusted to yield 5 × 10^8^ CFU/mL per strain for inoculation. Plating of cultures was conducted to confirm inoculation concentration.

### 2.2. Faecal Sample Preparation

Fresh faecal samples were provided by 3 healthy donors free from GIT disorders (2 male, 1 female), aged 21–24. Donors were not regular users of pre/probiotics or consumers of live yoghurt and had not consumed antibiotics in the 3 months prior to donating. Samples were collected and placed in an anaerobic jar using Thermo Scientific AnaeroGen 2.5 L anaerobic sachets (Oxoid, Basingstoke, UK). Samples were used for inoculation within 2 h of production. To form a 10% faecal slurry (*w*/*v*), 15 g of weighed faecal sample was homogenised with 135 mL of anaerobic PBS for 2 min using a stomacher (Stomacher 400, Seward, West Sussex, UK) at 240 paddle beats/min.

### 2.3. Batch Culture Fermentation

pH controlled, anaerobic, stirred batch cultures were performed in triplicate, with a sample from a different faecal donor used for each experiment. First, 135 mL of standard basal nutrient medium [28] with additional 0.1% tryptone (0.15 g) and 0.2% lactose (0.3 g) for bacteria growth was steamed and aseptically added to autoclaved 300 mL vessels. Vessels were then left to gas overnight using N_2_ at a rate of 15 mL/min to achieve anaerobic conditions.

Vessels were maintained at a temperature of 37 °C using a circulating water bath. The media were adjusted to pH 5.5 and subsequently maintained between 5.4 and 5.6 using pH controllers (Electrolab, Tewkesbury, UK) connected to 0.5 M solutions of HCL and NaOH. This pH was selected in order to mimic conditions of the proximal colon, under which GABA synthesis has previously been reported [29,30]. Immediately prior to faecal inoculation, overnight probiotic cultures were added to vessels to provide an estimated concentration of 5 × 10^8^ CFU. In addition, each fermentation run included a negative control vessel, to which only the faecal slurry was added, and a positive control vessel, to which inulin (Synergy 1, Beneo, Belgium) (1.5 g) was added as an additional substrate.

All vessels were inoculated with 15 mL of faecal slurry (10% *w*/*v*) to give a final concentration of 1% faeces (*w*/*v*). Baseline samples were taken immediately post-inoculation, and further samples were collected at 4, 8, 24, and 48 h; a stable pH and anaerobic conditions were maintained throughout.

### 2.4. Preparation of Samples

For Liquid Chromatography–Mass Spectroscopy (neurotransmitters), Gas Chromatography (short-chain fatty acids), and Fluorescence in situ Hybridisation (enumeration of bacteria), 1 mL, 1.5 mL, and 0.75 mL of sample were aliquoted to Eppendorfs, respectively; 1 mL samples were immediately stored at −20 °C. For GC, samples were centrifuged at 11, 600 g for 10 min before transferring the supernatant and storing the pellet at −20 °C. For FISH, samples were centrifuged at 11, 600 g for 5 min. After removing the supernatant, the pellet was resuspended in 375 μL of PBS before adding 1125 μL of 4% paraformaldehyde. These samples were then stored at 4 °C for 4–8 h before being washed twice with 1 mL of PBS and resuspending the pellet in 150 μL of PBS. Finally, 150 μL of ethanol was added, the samples were vortexed to homogenise, and then stored at −20 °C.

#### 2.4.1. Fluorescence In Situ Hybridisation with Flow Cytometry (Flow-FISH)

Preparation of samples followed the protocol of Grimaldi and colleagues [31]. Briefly, samples were removed from storage at −20 °C and vortexed to redisperse. Then, 75 μL of sample were suspended in 500 μL of PBS before vortexing and centrifuging for 3 min at 11,600× *g* (consistent for all centrifuging during this process). For permeabilisation of the bacterial cell wall, supernatant was discarded, and the pellet resuspended in TE-FISH containing lysozyme (1 mg/mL) and incubated in the dark for 10 min at room temperature. Samples were then re-centrifuged and washed using 500 μL PBS. For in situ hybridisation, pellets were resuspended in 150 μL of hybridisation buffer (0.9 M NaCl, 0.2 M Tris-HCl (pH 8.0), 0.01% sodium dodecyl sulphate, 30% formamide), centrifuged, and resuspended again in 1 mL. Then, 50 μL of this solution was added to each Eppendorf containing 4 μL of the oligonucleotide probe solutions, which were vortexed and incubated overnight at 35 °C using heating blocks. Following incubation, 125μL of hybridisation buffer was added, and Eppendorfs were vortexed and centrifuged as standard. After discarding the supernatant, pellets were resuspended in 175 μL of washing buffer (0.064 M NaCl, 0.02 M Tris/HCl (pH 8.0), 0.5 M EDTA (pH 8.0), 0.01% sodium dodecyl sulphate), vortexed to homogenise, and then incubated at 37 °C for 30 min in the heating block. The washed pellets were then centrifuged once again, resuspended in 300 μL of PBS, vortexed, and then stored in the dark at 4 °C ready for flow cytometry. Enumeration of bacteria was conducted using the Accuri C6 flow cytometer and analysed using the Accuri CFlow Sampler software.

Ten oligonucleotide probes (Table 1) were selected for inclusion, targeting a range of functionally relevant bacterial populations. Additionally, a mixed 338EUB probe was used to enumerate total bacteria.

#### 2.4.2. Gas Chromatography

Preparation of samples for GC was carried out in line with the method previously described by Richardson and colleagues [42]. Samples were defrosted, vortexed, and 1mL transferred to 100 mm × 16 mm glass vials, in addition to 50 µL internal standard (0.1 M 2-ethylbutyric acid) 0.5 mL concentrated HCl and 2 mL diethyl ether. Vials were vortexed for 1 min and centrifuged for 10 min at 2000 g (Eppendorf 5804 R, Stevenage UK). The upper diethyl ether layer was extracted and transferred to new vials, from which 400 µL were taken and added with 50 µL of MTBSTFA to screwcap HPLC vials. The vials were protected from light and stored at room temperature for 72 h prior to analysis to allow for all SCFAs, including lactate, to derivatise.

Samples were analysed using a 5690 series Gas Chromatograph (Hewlett Packard, London, UK) with HP-5 ms column (L × I.D. 30 m × 0.25 mm, 0.25 μm film thickness) coating of crosslinked (5%-phenyl)-methylpolysiloxane (Agilent, Santa Clara, CA, USA). Then, 1 μL of each sample was injected with a run time of 17.7 min. Injector and detector temperatures were 275 °C and the column temperature programmed from 63 °C to 190 °C at 5 °C per min and held at 190 °C for 30 min. Helium was used as the carrier gas at a flow rate of 1.7 mL/min (head pressure, 133 KPa). The external standard solution included acetic acid (30 mM); propionic acid (20 mM); *n*-butyric acid (20 mM); *n*-valeric acid (5 mM); iso-butyric acid (5 mM); iso-valeric acid (5 mM) (all Sigma-Aldrich). Quality control (QC) samples of external standard solution were included between donors to maintain accurate calibration. Peak integration was performed using Agilent Chemstation software (Agilent Technologies, Cheadle, UK), and quantification of each SCFA (mM) was calculated using internal response factors as described previously [43].

#### 2.4.3. Liquid Chromatography–Mass Spectroscopy

Samples were first removed from storage at −20 °C and centrifuged for 5 min at 2000 g. Then, 10 µL of supernatant was added to 9.99 mL of HPLC water to form a 1:1000 dilution, which was then filtered using 0.22 µm syringe filters. Then, 1 mL was added to a screwcap HPLC vial for analysis. In addition, 1 mL of batch culture medium was prepared in the same manor for analysis as a control. Individual stock solutions were prepared using analytical standards powders of dopamine hydrochloride (≥99%, Alfa Aesar (Lancashire, UK)), serotonin (≥98%, Sigma-Aldrich), tryptophan (≥98%, Sigma-Aldrich), GABA (≥99%, Sigma-Aldrich), L(-)-epinephrine (≥99%, Acros Organics (Geel, Belgium)), L-noradrenaline (≥98%, Alfa Aesar), and kynurenic acid (≥98%, Sigma-Aldrich), each at 10,000 ng/mL. A mixed standard solution was then prepared from the individual stock solutions and used to create a 7 level calibration series with the following dilutions: 10, 5, 1, 0.5, 0.25, 0.125, and 0.0625 ng/mL. Additionally, a 1 ng/mL standard was run every 20 samples as a QC.

Samples were analysed by liquid chromatography–mass spectrometry/mass spectrometry (LC–MS/MS) using an Agilent 1200 HPLC system attached to a 6410 triple-quadrupole mass spectrometer with electrospray ion source in positive ion mode. A gradient separation was carried out using a 150 × 2.1 mm Discovery HS F5—3 column, with a 2 × 2.1 mm Discovery C18 Supelguard precolumn (both 3 μm particle size; Supelco, Poole, UK). The column was maintained at 40 °C. Mobile phase A was 0.1% formic acid in water and mobile phase B was 0.1% formic acid in acetonitrile. The column flow rate was maintained at 0.4 mL/min. The timetable was as follows: 0–2 min, 100% A; 5 min, 75% A; 11 min, 65% A; 15–20 min 5% A; 20.1–30 min, 100% A. The injection volume was 25 μL. The eluant from the column was run to waste from 0 to 1 min, and data were collected from 1 to 18 min. Data were acquired in dynamic MRM mode. The transitions studied and voltages used are shown in Table 2. Two transitions were acquired for each compound.

### 2.5. Statistical Analysis

All statistical analyses were performed using R statistical software [44]. The effect of time (0, 8, and 24 h of fermentation) and vessel (negative control, positive control (inulin), *B. coagulans*, *B. subtilis*, *L. reuteri*, *Lc. lactis*, *L. rhamnosus*, *P. freudenreichii*) on specific bacterial groups, SCFAs, and neurotransmitters was assessed using repeated-measures two-way ANOVAs with post-hoc pairwise comparisons (Bonferroni corrected). As inulin is known to affect SCFA production, particularly acetate and lactate, it was anticipated that change in SCFA concentration over the fermentation period would be greatest in the positive control vessel. Given that inulin was only used as a positive control substrate in this model and that the effect of inulin on metabolite production is not relevant to the aims of this work, statistical analysis of SCFA concentration was run both including and excluding the positive control vessel, in case the larger known effect of inulin on SCFA concentration masked any smaller effects in the probiotic vessels of interest. Statistical significance was set to *p* < 0.05 and data are presented as mean ± standard error unless otherwise stated.

## 3. Results

### 3.1. Enumeration of Bacteria with Flow-FISH

**Figure 1:**
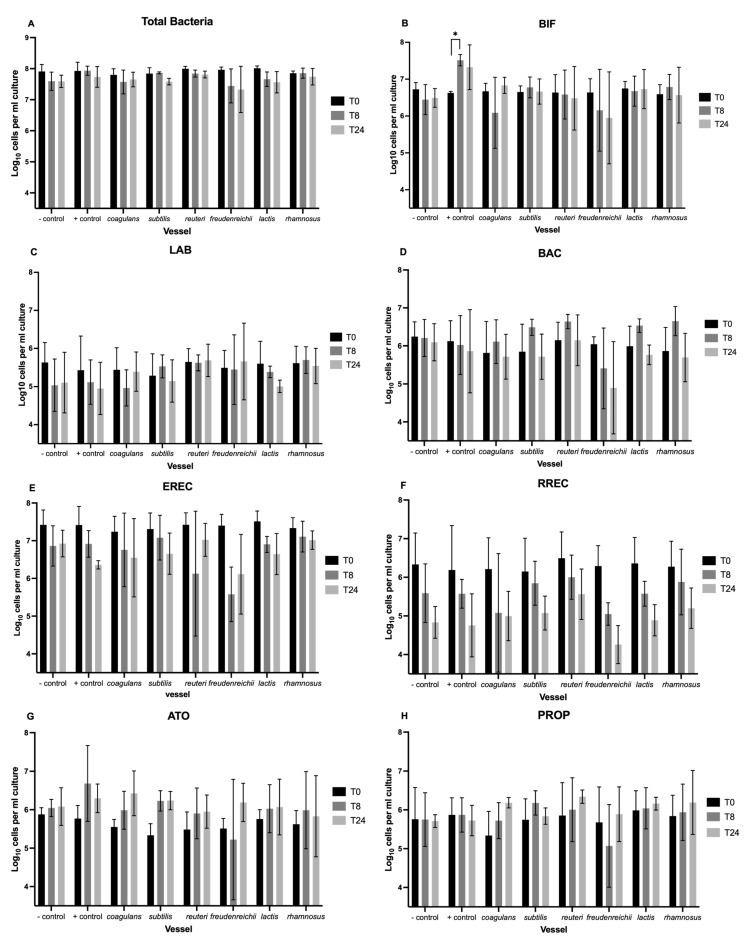

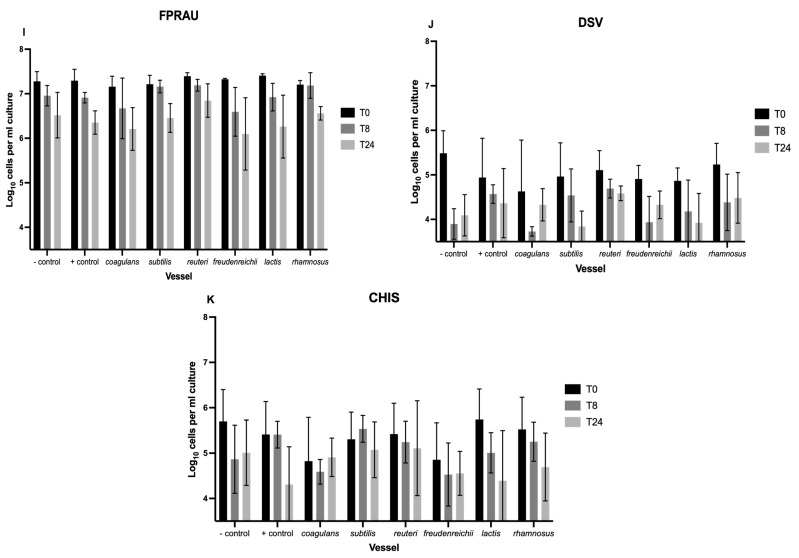
Enumeration of bacteria by Flow-FISH at baseline (T0) and following 8 (T8) and 24 (T24) hours of faecal (1%) fermentation within the negative control, positive control, and six probiotic vessels, represented as log_10_ cells/mL culture. Target bacteria included: total bacteria (**A**), *Bifidobacterium* spp. (BIF) (**B**), *Lactobacillus* spp. (LAB) (**C**), most Bacteroidaceae and Prevotellaceae (BAC) (**D**), *Clostridium coccoides–Eubacterium rectale* group (EREC) (**E**), *Roseburia* subcluster (RREC) (**F**), *Faecalibacterium prausnitzii* (FPRAU) (**G**), *Clostridium* cluster IX (PROP) (**H**), *Atopobium–Coriobacterium* spp. (ATO) (**I**), Desulfovibrio (DSV) (**J**), and *Clostridium histolyticum* (CHIS) (**K**). Values are presented as mean ± standard error from three independent experiments. Significant change within vessels is indicated as * *p* < 0.05. No significant difference between the negative control and other vessels was observed at any of the sampling timepoints.

Figure 1 illustrates change in bacterial groups between baseline (T0), 8 h (T8), and 24 h (T24). No significant difference in bacterial numbers was found between vessels at baseline. A significant main effect of time was observed on total bacteria, and most bacterial groups assessed, including *Clostridium coccoides–Eubacterium rectale* (EREC), *Roseburia* subcluster (RREC), *Faecalibacterium prausnitzii* (FPRAU), Desulfovibrio (DSV), and *Clostridium histolyticum* (CHIS), showed that bacterial numbers steadily declined over the 24-h period across all probiotic and non-probiotic vessels (all *p* < 0.05) (Figure 1). In comparison, no main effect of time or vessel was observed for numbers of *Bacteroides-Prevotella* spp. (BAC) (Figure 1D) or *Clostridium* cluster IX (PROP) (Figure 1H). However, in contrast to other bacteria groups, visual inspection of the data indicates that numbers of *Bacteroides-Prevotella* spp. increased between T0 and T8 across all probiotic vessels (except P. freudenreichii), but not in the control vessels. Similarly, numbers of *Clostridium* cluster IX displayed a general increase in the probiotic vessels over the fermentation period when compared to the control vessels, although these changes were non-significant.

With regards *to Bifidobacterium* spp. (BIF), a time by vessel interaction was observed (F(14,28) = 2.068, *p* = 0.049). Pairwise comparisons indicate that this was driven by a significant increase from 6.6 to 7.5 log10 cells/mL by T8 in the positive control vessel, following the fermentation of inulin (*p* = 0.021) (Figure 1B). No significant change in *Lactobacillus* spp. (LAB) or *Atopobium–Coriobacterium* spp. (ATO) was found.

### 3.2. Short-Chain Fatty Acids

**Figure 2:**
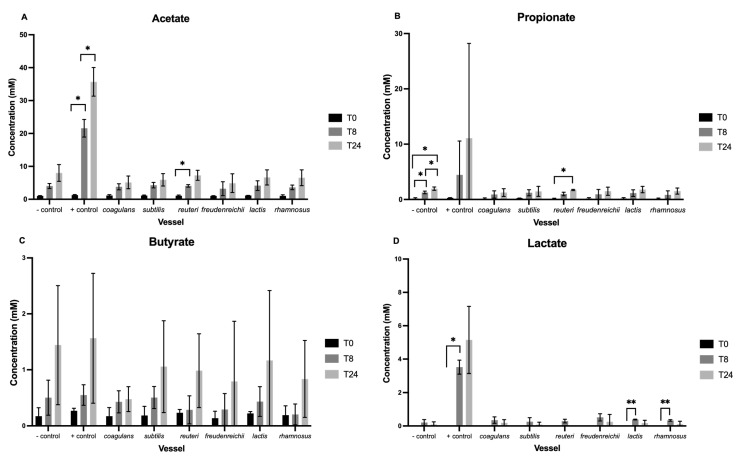
SCFA concentrations of acetate (**A**), propionate (**B**), butyrate (**C**), and lactate (**D**) (mM) per vessel (excluding the positive control vessel) at baseline and following 8 (T8) and 24 h (T24) of fermentation. Values are mean ± standard error. Significant change within vessels is indicated as * *p* < 0.05 and ** *p* < 0.01. No significant difference between the negative control and other vessels was observed at any of the sampling timepoints.

Figure 2 demonstrates changes in SCFA concentration over the course of fermentation. No significant difference between vessels at baseline was found. Levels of valerate, iso-valerate, and iso-butyrate were below that of minimum detection and are therefore not presented.

**Figure 3:**
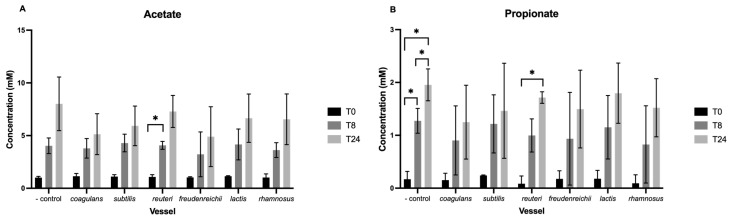

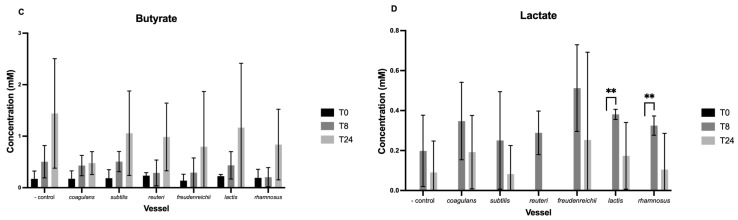
SCFA concentrations of acetate (**A**), propionate (**B**), butyrate (**C**), and lactate (**D**) (mM) per vessel (excluding the positive control vessel) at baseline and following 8 (T8) and 24 h (T24) of fermentation. Values are mean ± standard error. Significant change within vessels is indicated as * *p* < 0.05 and ** *p* < 0.01. No significant difference between the negative control and other vessels was observed at any of the sampling timepoints.

Looking at acetate (Figure 2A), there was a significant main effect of time (F(1,68) = 24.66, *p* < 0.001), substrate (F(1,68) = 10.2, *p* = 0.002), and time by substrate interaction (F(1,68) = 5.94, *p* = 0.017). Pairwise comparisons highlight a significant increase from T0 to T8 (*p* < 0.05) and T0 to T24 (*p* < 0.05) in the positive control vessel, in addition to a significant increase from T0 to T8 following the addition of *L. reuteri* (*p* < 0.05). After exclusion of the positive control vessel (Figure 3A), only the main effect of time was maintained, where concentration increases over the 24-h period across all vessels (F(2,42) = 68.36, *p* < 0.001). No change in pairwise comparisons was observed.

For propionate, a significant main effect of time was observed (F(1,68) = 6.254, *p* = 0.015) only (Figure 3B). Pairwise comparisons indicate this increase in concentration is significant from T0 to T8 (*p* < 0.05), T8 to T24 (*p* < 0.05), and T0 to T24 (*p* < 0.05) in the negative control vessel. Additionally, concentration significantly increased between T0 and T24 following the addition of *L. reuteri* (*p* < 0.05). The main effect of time (F(2,42) = 44.55, *p* < 0.001) and all post-hoc effects were maintained when excluding the positive control vessel.

Concentration of butyrate increased over the 24-h period across all vessels, reflected as a significant main effect of time (F(1,68) = 32.86, *p* < 0.001) (Figure 3C). However, no main effect of substrate or interaction was observed.

Concentration of lactate increased across all vessels by T8 and fell by T24 (Figure 2D). Main effects of time (F(1,68) = 5.13 *p* = 0.027) and substrate (F(1,68) = 6.38, *p* = 0.014) were significant, while their interaction was bordering on significant (F(1,68) = 3.92, *p* = 0.052). Pairwise comparisons indicate a significant increase in concentration from T0 to T8 in the positive control vessel (*p* < 0.05) and following the addition of *Lc. lactis* (*p* < 0.01) and *L. rhamnosus* (*p* < 0.05). When excluding the positive control vessel, the main effect of time (F(2,42) = 23.22, *p* < 0.001) and pairwise comparisons remain (Figure 3D).

### 3.3. Neurotransmitters

**Figure 4:**
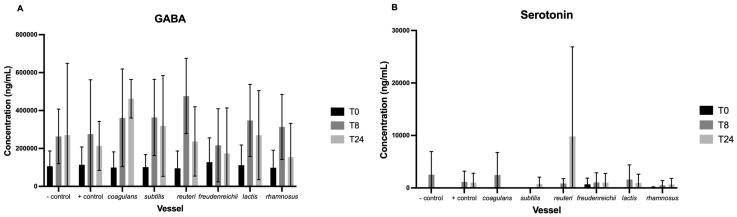

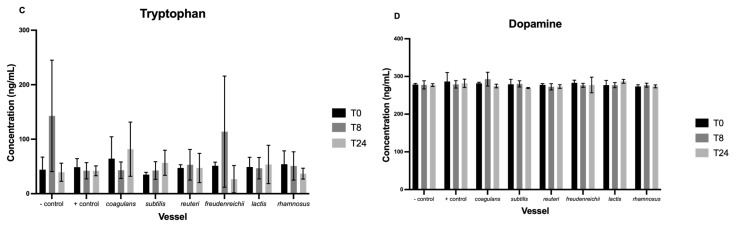
Concentrations of GABA (**A**), serotonin (**B**), tryptophan (**C**), and dopamine (**D**) (mM) per vessel (excluding the positive control vessel) at baseline and following 8 (T8) and 24 h (T24) of fermentation. Values are mean ± standard error. No significant difference within or between vessels was observed at any of the sampling timepoints.

Changes in neurotransmitter concentrations are illustrated in Figure 4. No significant difference in baseline concentration was detected between vessels for each compound. Levels of epinephrine, norepinephrine, and kynurenic acid were below that of minimum detection and are not presented.

The fermentation process elicited a significant main effect of time on GABA concentration (F(1,68) = 8.63, *p* = 0.005). Pairwise comparisons reveal that the increase in concentration from T0 to T8 was trending towards significance (*p* < 0.1) following the addition of *L. reuteri*, *Lc. Lactis*, and *L. rhamnosus*, and from T0 to T24 in the vessel with added *B. coagulans* (Figure 4A). No other statistically significant changes in neurotransmitter production were observed.

## 4. Discussion

This work aimed to assess the production of neuroactive metabolites in faecal microbiota under physiologically relevant conditions, and to explore the additional impact of several probiotic bacteria on both the faecal bacterial community and metabolite production using pH controlled, anaerobic in vitro batch culture models. In addition to a negative control vessel, which allowed for comparison of the probiotic vessels to the natural microbiota, inulin was included as a positive control substrate due to its known effects on *Bifidobacterium* spp. and SCFA production [27,45,46]. As expected, fermentation of inulin resulted in a substantial increase in *Bifidobacterium* spp., coupled with significantly increased concentrations of acetate and lactate over the 24-h period. These results are in line with previous data describing a bifidogenic effect of inulin, and therefore provide evidence that the batch culture fermentation models functioned as intended. 

Batch culture fermentation models allowed the detection of GABA, serotonin, tryptophan, and dopamine under conditions relevant to the human GIT. Whereas previous work has typically employed optimal pH, temperature, and growth mediums when reporting the presence/production of neurotransmitters by isolated bacteria strains [19,47,48], the current work demonstrated neurotransmitter production in human faecal microbiota when under physiologically relevant conditions, using a standard basal media, in the absence of colonic cells. As such, the current data provide strong evidence for the bacterial derivation of these four metabolites under conditions relevant to the human GIT.

This is perhaps unsurprising with regards to GABA. GABA is synthesised through the decarboxylation of L-glutamate by glutamic acid decarboxylase (GAD), a system which has been established in several bacteria strains to provide a protective mechanism against the acidic gut environment; hence, GABA synthesis has been found to be highest at low pH [29]. However, the presence of tryptophan, serotonin, and dopamine under these conditions is more novel, and at present is it unclear how enteric bacteria may mediate and/or produce these neuroactive metabolites. Serotonin synthesised in isolated bacterial cultures has been speculated to occur in the same manner as seen in plants, via the decarboxylation of tryptophan into tryptamine [49]. The gut microbiota also appears to mediate how dietary tryptophan is metabolised into its various derivatives, such as indole, kynurenine, and serotonin [50,51], but microbial production of tryptophan and dopamine is not yet understood. As such, future work to further elucidate the precise mechanism(s) of production is necessary. While the detection of these metabolites in the current fermentation models suggests some level of bacterial derivation under relevant conditions, the concentrations of serotonin, tryptophan, and dopamine were relatively low compared to that of GABA. This implies that while there may be bacteria with the capacity to synthesise these compounds, human intestinal cells are likely required in these production pathways to produce physiologically relevant quantities in the host. For example, gut microbiota may mediate the biosynthesis of serotonin by influencing the expression of tryptophan hydroxylase 1 (TPH1)—a rate limiting step in the synthesis of serotonin—in enterochromaffin cells, where the majority of host serotonin is located and transferred to the periphery [52]. As such, while suitable for exploring levels of microbially derived GABA, batch culture fermentation models may not provide an optimal method for the exploration of other neuroactive compounds, such as serotonin, that likely require the provision of cells. That said, it should be noted that the present batch cultures were purposely maintained at a pH comparable to that of the proximal colon to stimulate GABA production, but this pH may not be optimal for the utilisation and production of other neurotransmitters and more alkaline pH, such as that found in the transverse or distal colon, which may elicit different results. Modelling of the transverse and distal areas of the colon may also be beneficial when exploring neuroactive metabolite production as the vagus nerve is believed to have afferent nerve interactions with both regions, providing a potential gut-brain pathway [53].

In addition to assessing the potential for microbially derived neuroactive metabolites in the GIT, this work explored the effect of additional probiotic bacteria on both microbiota composition and metabolite production. With regards to microbiota composition, the selected probiotic bacteria did not result in a significant shift in log_10_ cells/mL for any bacteria group assessed, including *Lactobacillus* spp., over the fermentation period. This is perhaps unsurprising given the abundance of faecal bacteria relative to the quantity of probiotic bacteria added per mL (3.3 × 10^6^ CFU). As batch cultures provide a closed-loop, an anaerobic environment with a limited supply of nutrients, a steady decline in bacterial numbers may be expected due to depletion of nutrients present in the basal medium. Flow FISH results indicate that this was the case for total bacteria and across most bacteria groups assessed. In comparison, numbers of *Bacteroides-Prevotella* spp. and *Clostridium* cluster IX were maintained and appear to gradually increase following the addition of *B. coagulans*, *B. subtilis*, *L. reuteri*, *Lc. Lactis*, and *L. rhamnosus* over 8 and 24 h, respectively, when compared to the control vessels. While this difference in trajectory suggests these strains may facilitate the maintenance and/or growth of these specific bacteria groups, log_10_ increases from baseline were not significant within these probiotic vessels, nor statistically different to numbers in the negative control vessel.

Concentrations of acetate, propionate, and butyrate increased over the fermentation period across all vessels, while concentrations of lactate increased by 8 h and fell once again by 24 h. This general increase in SCFA production over the fermentation period is likely due to fermentation of the lactose and tryptone within the basal media present in all vessels. On the other hand, the fall in lactate between 8 and 24 h is likely a reflection of important cross-feeding pathways, where certain bacteria are able to utilise lactate for the production of other SCFAs and metabolites [54]. This fall in concentration would not be expected for other SCFAs present within this closed environment, as they are broken down less readily than lactate. With the exception of the positive control vessel (inulin), synthesis of lactate was greatest in the *Lc. lactis* and *L. rhamnosus* vessels, where concentrations significantly increased from baseline after 8 h. Both species are known lactic acid producing bacteria (LAB), and their ability to produce lactic acid has previously been confirmed in vitro [55,56]. The current data not only provide evidence of enhanced lactic acid production under physiologically relevant conditions, but also highlight that probiotic bacterium such as *Lc. lactis* and *L. rhamnosus* are able to interact with existing host bacteria to influence metabolite production without necessarily causing a quantitative shift in bacterial composition.

Microbially derived lactic acid has been linked to several health benefits, including lowering cholesterol, anti-inflammatory properties, and increased nutrient absorption from diet [57]. Additionally, as mentioned previously, lactic acid is involved in the production of other SCFAs such as acetate, butyrate, and propionate. For example, lactate can be converted to propionate via the acrylate pathway by select Firmicutes [58] or via the succinate pathway, primarily by Bacteroidetes [53]. Many commensal species have the ability to convert lactate into acetate via acetyl-CoA [59], while select bacteria, such as *Eubacterium hallii* strains, are able to produce butyrate through the butyryl-CoA:acetate-CoA transferase route [60]. As such, increasing the availability of lactate may subsequently increase synthesis of other beneficial SCFAs. This may be significant in the context of the microbiota-gut-brain axis, as SCFAs play a role in the synthesis of various neuroactive metabolites and neurotransmitters [25,26,61]. In addition, SCFAs support gut barrier function and immune function, which in turn may improve tryptophan availability for serotonin [62]. However, previous work suggests that while pH 5.5 is supportive for the production of lactate by LAB, it does not provide an optimal environment for lactate-utilising bacteria and can led to a detrimental accumulation of lactic acid [11,63]. As the current fermentation models were maintained at pH 5.5, we perhaps would not expect a significant increase in lactate to be reflected as an increase in the concentrations of other SCFAs.

Although there were no statistically significant effects observed of the selected probiotic strains on neurotransmitter production, trends in the data suggest that *L. reuteri*, *Lc. Lactis*, *L. rhamnosus*, and *B. coagulans* may help to enhance the production of GABA. Production of GABA has typically been associated with LAB bacteria, and previous work has found species including *Lc. lactis* and *B. coagulans* to be good candidates for GABA synthesis due to the expression of GAD system genes [64,65]. Additionally, species such as *L. rhamnosus* are being actively investigated for their potential GABAergic effect on mental and cognitive health disorders, with promising effects in animal models, particularly for depression [65]. However, it is important to note that there is currently no evidence that gut-derived neurotransmitters cross the blood brain barrier, and there is little understanding as to the mechanisms via which gut-derived neurotransmitters may affect the brain.

It is also of importance to highlight limitations to the current work. Batch culture models provide a closed system with an equal amount of carbon and nitrogen for bacteria to grow on within each vessel, and the use of a negative control vessel allows for undigested food sources within the faeces to be ruled out as responsible for changes over the fermentation period. As such, we can be confident that the results are a true reflection of microbial fermentation and that any changes in the active vessels can be attributed to the additional pre- or probiotics. However, the three faecal donors in this study elicited substantial inter-donor variability in both bacterial composition and metabolite production (see Supplementary Materials). As a result, the ability to observe statistically significant change in these parameters may have been compromised, making it more difficult to establish the effects of the select probiotic strains. As such, determining which microbial members are involved in these changes and how different starting consortium of bacteria interact with the effect of probiotics is an important avenue of future work. With that said, in vitro batch cultures performed in triplicate do provide valuable data that matches well with the outcomes of intervention studies, and this is exemplified in the current experiment by the bifidogenic effect seen in the positive control vessel which is supported by the results of in vivo work [66]. In addition, although the abundance of SCFAs matched that as found in vivo with acetate being most abundant, followed by propionate and butyrate in similar quantities, concentration of SCFAs in these models were generally lower than expected compared to previous work [67,68]. It is likely, therefore, that the lactose content in these batch cultures was too low to support greater production.

To conclude, the present work provides evidence for the production of several neurotransmitters in the absence of colonic cells while under physiologically relevant conditions, suggesting bacterial derivation of these neuroactive metabolites. However, relatively low concentrations of tryptophan, serotonin and dopamine, compared to GABA, suggest that bacterial synthesis may not provide a primary production pathway for these metabolites, and instead colonic cells may be required to reach physiologically relevant levels. The addition of probiotic bacteria did not lead to significant shifts in microbiota composition, but trends in the current data suggest they may support the growth of *Bacteroides-Prevotella* spp. and *Clostridium* cluster IX and could enhance concentrations of microbially derived GABA. In addition, *Lc. lactis* W58 and *L. rhamnosus* W198 led to significantly increased concentrations of lactate after 8 h of fermentation. As such, the trends in the current data warrant further exploration to better understand how these probiotic strains may influence cognitive and psychological behaviour via microbially derived metabolites and the gut-brain axis. Future work may wish to model these effects using more comprehensive gut models [69] that allow for the provision of more nutrients and the ability to assess metabolite production at a range of physiologically relevant pHs mimicking different regions of the human colon.

## Figures and Tables

**Table 1:** Oligonucleotide probe sequences and corresponding target species.

Probe	Sequence	Target Species
Non-Eub	ACTCCTAGGGAGGCAGA	Control probe for EUB338 [32]
Eub338I+	GCTGCCTCCCGTAGGAGT	Most bacteria [33]
Eub338II+	GCAGCCACCCGTAGGTGT	*Planctomycetales* [33]
Eub338III+	GCTGCCACCCGTAGGTGT	*Verrucomicrobialesm* [33]
Bif164	CATCCGGCATTACCACCC	*Bifidobacterium* spp. [34]
Lab158	GGTATTAGCAYCTGTTTGGA	*Lactobacillus* and *Enterococcus* [35]
Bac303	CCAATGTGGGGGACCTT	*Bacteroidaceae*, *Prevotellaceae* [36]
Erec482	GCTTCTTAGTCARGTACCG	Most of the *Clostridium coccoides-Eubacterium rectale* group [37]
Rrec584	TCAGACTTGCCGYACCGC	*Roseburia* [38]
Ato291	GGTCGGTCTCTCAACCC	*Atopobium* cluster [39]
Prop853	ATTGCGTTAACTCCGGCAC	*Clostridium* cluster IX [38]
Fprau655	CGCCTACCTCTGCACTAC	*Faecalibacterium prausnitzii* and relatives [40]
DSV687	TACGGATTTCACTCCT	*Desulfovibrio* genus [41]
Chis150	TTATGCGGTATTAATCTYCCTTT	Most of the *Clostridium histolyticum* group [37]

**Table 2:** LC-MS/MS conditions used for quantification in faecal supernatant.

Compound	Retention Time (Min)	Retention Time Window (Min)	Precursor Ion (*m*/*z*)	Product Ion (*m*/*z*)	Fragment Or (V)	Collision Energy (V)	Classification
GABA	1.90	3	104	87	50	4	Organic acid
		3	104	45	50	20	
Norepinephrine	2.50	3	152	107	116	16	Catecholamine
			152	77	116	30	
Epinephrine	4.60	3	184	166	70	8	Catecholamine
			184	107	70	24	
Dopamine	7.00	3	154	137	75	8	Catecholamine
			154	91	75	28	
Serotonin	9.70	3	177	160	45	4	Amino acid derivative
			177	115	45	30	
Kynurenic acid	9.77	3	190	144	100	16	Organic acid
			190	172	100	4	
Tryptophan	10.20	3	205	188	78	4	Amino acid
			205	146	78	20	

## Supplementary Materials

The following supporting information can be downloaded at: https://www.mdpi.com/article/10.3390/nu15112563/s1, Figure S1. Concentration (mM) of acetate at baseline and following 8 and 24 h of fermentation per donors 1, 2 & 3 (left to right). Figure S2. Concentration (mM) of propionate at baseline and following 8 and 24 h of fermentation per donors 1, 2 & 3 (left to right). Figure S3. Concentration (mM) of butyrate at baseline and following 8 and 24 h of fermentation per donors 1, 2 & 3 (left to right). Figure S4. Concentration (mM) of lactate at baseline and following 8 and 24 h of fermentation per donors 1, 2 & 3 (left to right). Figure S5. Concentration (mM) of acetate at baseline and following 8 and 24 h of fermentation per donors 1, 2 & 3 (left to right). Figure S6. Concentration (mM) of propionate at baseline and following 8 and 24 h of fermentation per donors 1, 2 & 3 (left to right). Figure S7. Concentration (mM) of butyrate at baseline and following 8 and 24 h of fermentation per donors 1, 2 & 3 (left to right). Figure S8. Concentration (mM) of lactate at baseline and following 8 and 24 h of fermentation per donors 1, 2 & 3 (left to right). Figure S9. Concentration (ng/mL) of GABA at baseline and following 8 and 24 h of fermentation per donors 1, 2 & 3 (left to right). Figure S10. Concentration (ng/mL) of serotonin at baseline and following 8 and 24 h of fermentation per donors 1, 2 & 3 (left to right). Figure S11. Concentration (ng/mL) of tryptophan at baseline and following 8 and 24 h of fermentation per donors 1, 2 & 3 (left to right). Figure S12. Concentration (ng/mL) of dopamine at baseline and following 8 and 24 h of fermentation per donors 1, 2 & 3 (left to right). Table S1. Donor 1, Enumeration of bacteria for by Flow-FISH at baseline (0) and following 8 and 24 h of fermentation within the negative control, positive control, and six probiotic vessels, represented as log10 cells/mL culture. Target bacteria: *Bifidobacterium* spp.(BIF), *Lactobacillus* spp. (LAB), most Bacteroidaceae and Prevotellaceae (BAC), Clostridium coccoides–Eubacterium rectale group (EREC), Roseburia subcluster (RREC), Faecalibacterium prausnitzii (FPRAU), Clostridium cluster IX (PROP), Atopobium-*Coriobacterium* spp. (ATO), Desulfovibrio (DSV) and Clostridium histolyticum (CHIS). Table S2. Donor 2, Enumeration of bacteria for by Flow-FISH at baseline (0) and following 8 and 24 h of fermentation within the negative control, positive control, and six probiotic vessels, represented as log10 cells/mL culture. Target bacteria: *Bifidobacterium* spp.(BIF), *Lactobacillus* spp. (LAB), most Bacteroidaceae and Prevotellaceae (BAC), Clostridium coccoides–Eubacterium rectale group (EREC), Roseburia subcluster (RREC), Faecalibacterium prausnitzii (FPRAU), Clostridium cluster IX (PROP), *Atopobium-Coriobacterium* spp. (ATO), Desulfovibrio (DSV) and Clostridium histolyticum (CHIS). Table S3. Donor 3, Enumeration of bacteria for by Flow-FISH at baseline (0) and following 8 and 24 h of fermentation within the negative control, positive control, and six probiotic vessels, represented as log10 cells/mL culture. Target bacteria: *Bifidobacterium* spp.(BIF), *Lactobacillus* spp. (LAB), most Bacteroidaceae and Prevotellaceae (BAC), Clostridium coccoides–Eubacterium rectale group (EREC), Roseburia subcluster (RREC), Faecalibacterium prausnitzii (FPRAU), Clostridium cluster IX (PROP), *Atopobium-Coriobacterium* spp. (ATO), Desulfovibrio (DSV) and Clostridium histolyticum (CHIS).
