# Supplementary material for: The Effect of Probiotic Bacteria on Composition and Metabolite Production of Faecal Microbiota Using In Vitro Batch Cultures
Source: Nutrients. 2023 May 30;15(11):2563. doi: 10.3390/nu15112563 (PMC10255927; doi:10.3390/nu15112563)
Supplement: Supplementary file 1 [file nutrients-15-02563-s001.zip › nutrients-2354447-supplementary.pdf]

Supplementary data

A. Short chain fatty acids (including positive control vessel)

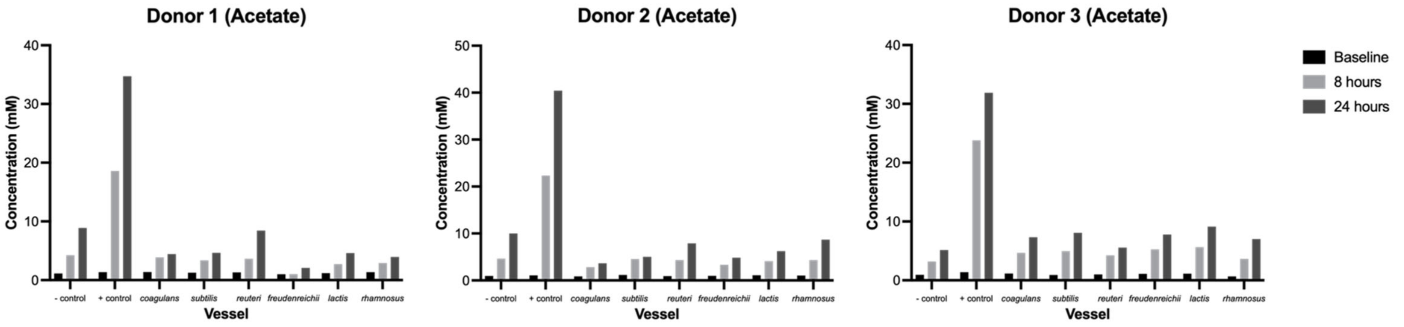

Figure S1. Concentration (mM) of acetate at baseline and following 8 and 24 hours of fermentation per donors 1, 2 & 3 (left to right).

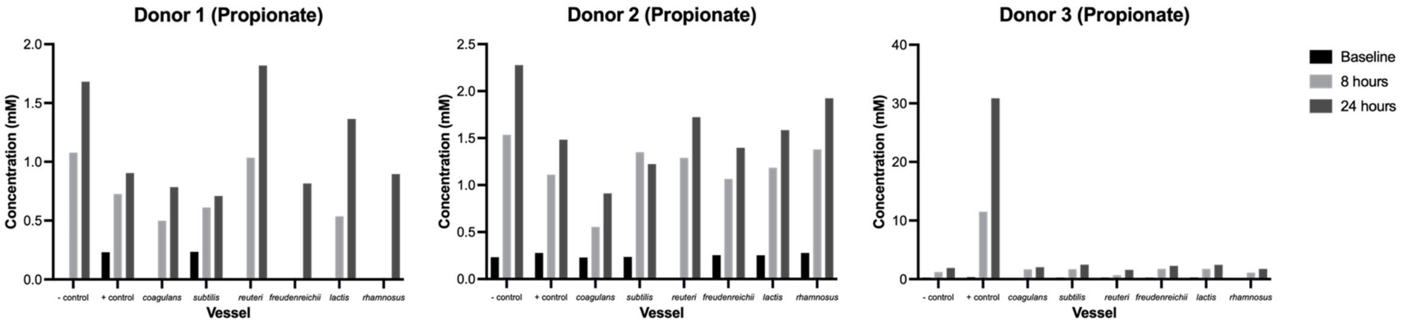

Figure S2. Concentration (mM) of propionate at baseline and following 8 and 24 hours of fermentation per donors 1, 2 & 3 (left to right).

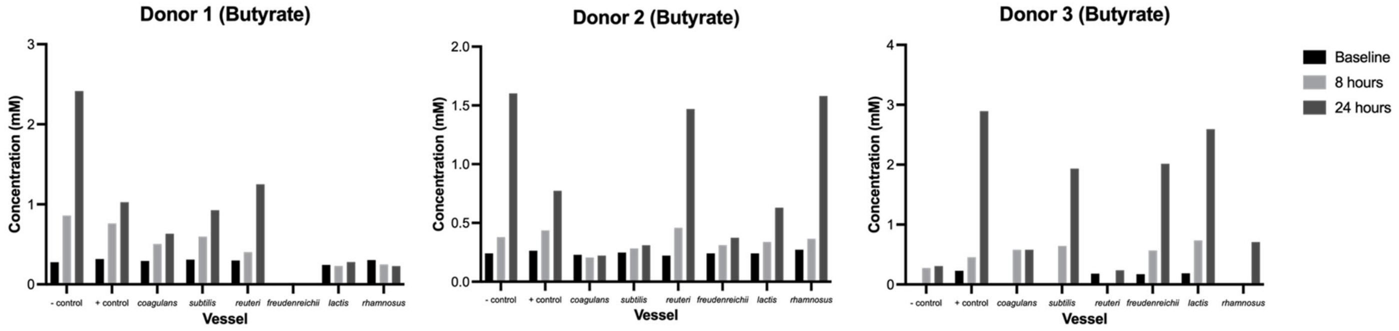

Figure S3. Concentration (mM) of butyrate at baseline and following 8 and 24 hours of fermentation per donors 1, 2 & 3 (left to right).

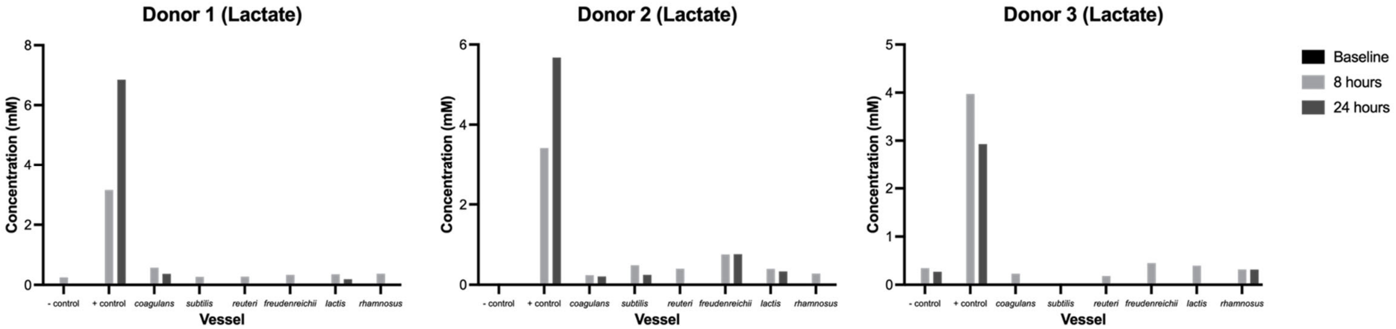

Figure S4. Concentration (mM) of lactate at baseline and following 8 and 24 hours of fermentation per donors 1, 2 & 3 (left to right).

B. Short-chain fatty acids (excluding positive control vessel)

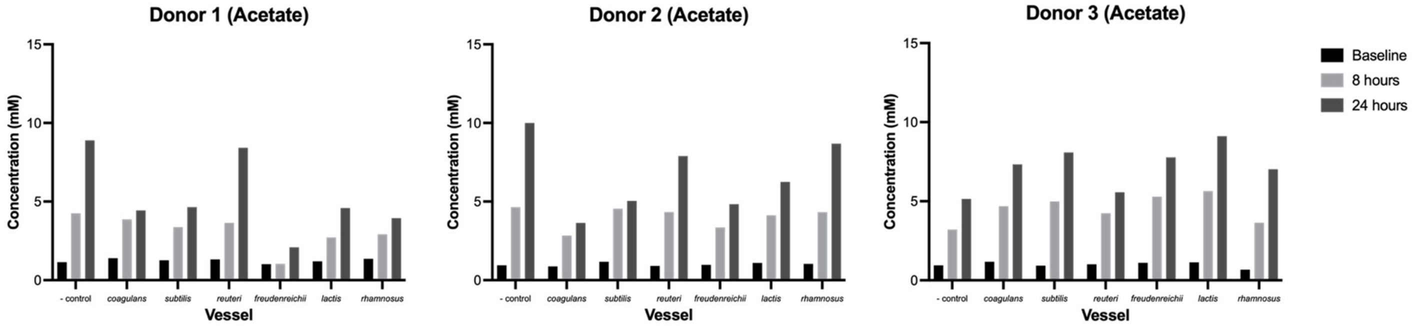

Figure S5. Concentration (mM) of acetate at baseline and following 8 and 24 hours of fermentation per donors 1, 2 & 3 (left to right).

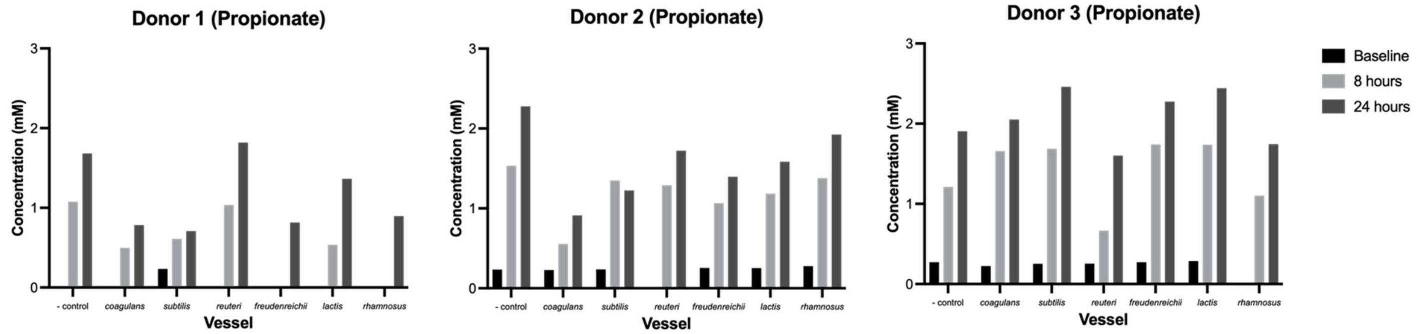

Figure S6. Concentration (mM) of propionate at baseline and following 8 and 24 hours of fermentation per donors 1, 2 & 3 (left to right).

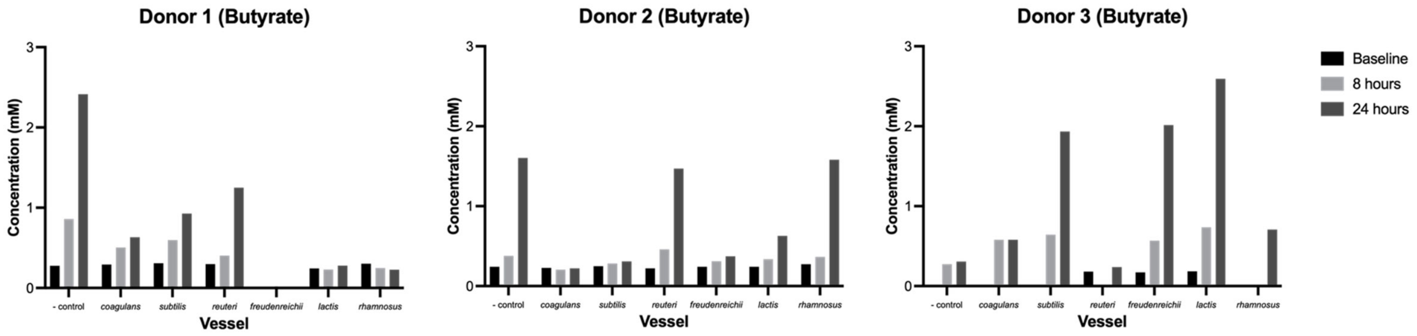

Figure S7. Concentration (mM) of butyrate at baseline and following 8 and 24 hours of fermentation per donors 1, 2 & 3 (left to right).

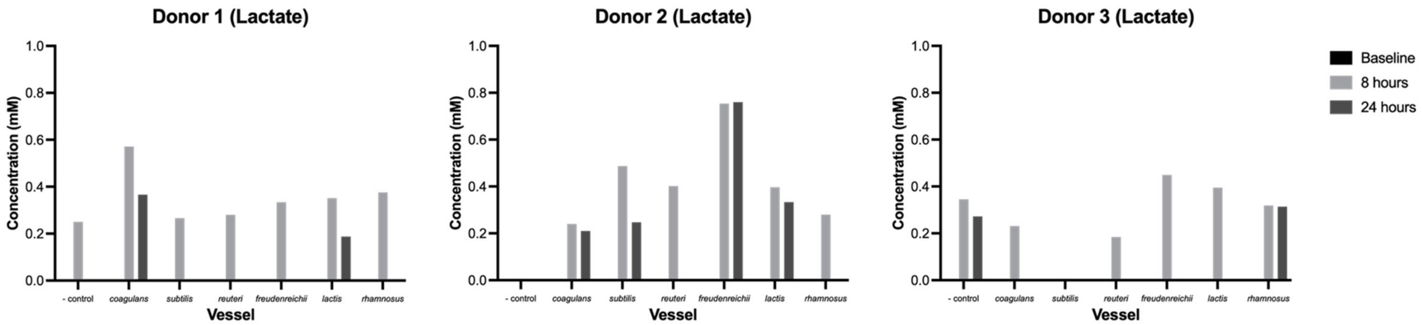

Figure S8. Concentration (mM) of lactate at baseline and following 8 and 24 hours of fermentation per donors 1, 2 & 3 (left to right).

C. Neurotransmitters

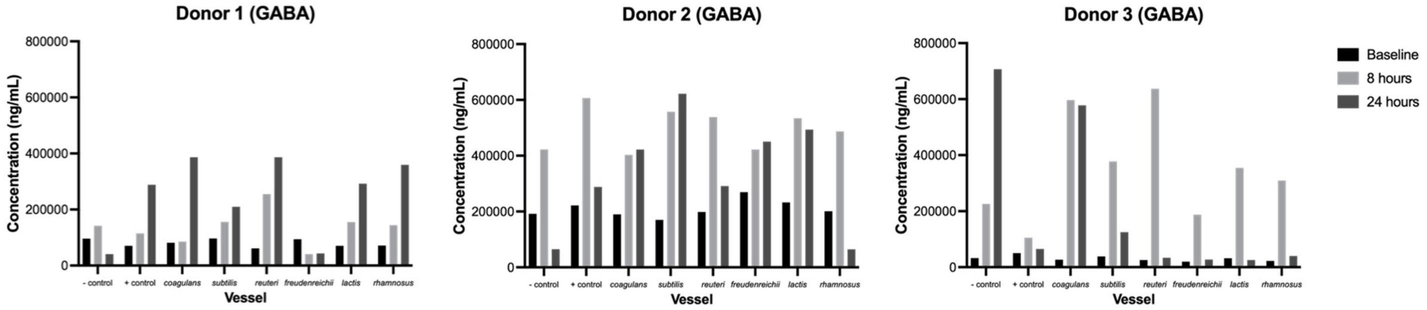

Figure S9. Concentration (ng/mL) of GABA at baseline and following 8 and 24 hours of fermentation per donors 1, 2 & 3 (left to right).

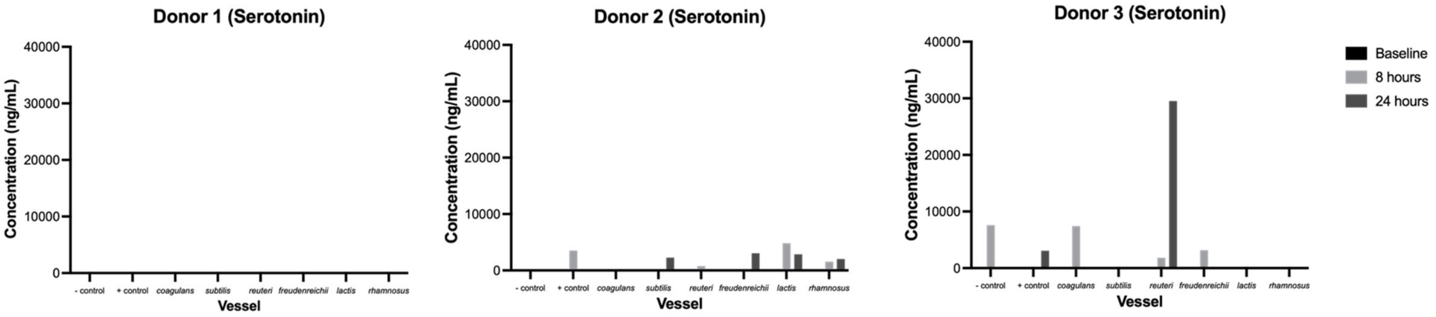

Figure S10. Concentration (ng/mL) of serotonin at baseline and following 8 and 24 hours of fermentation per donors 1, 2 & 3 (left to right).

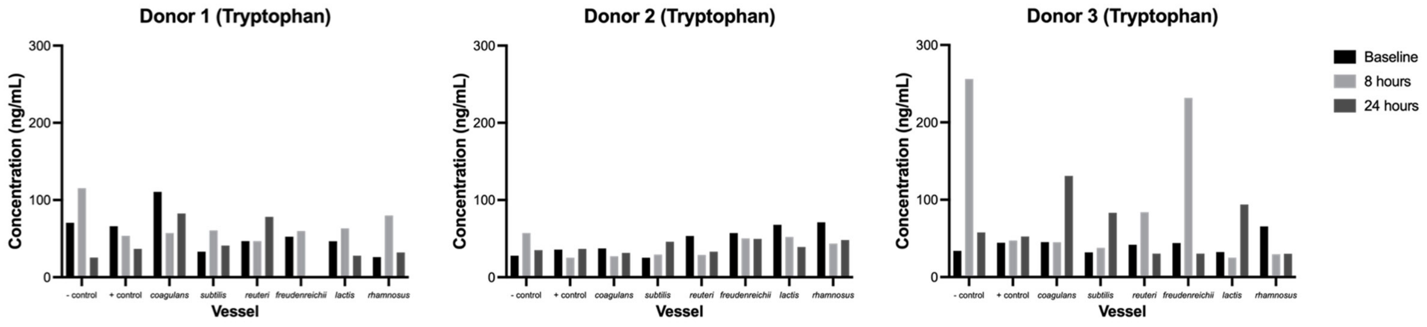

Figure S11. Concentration (ng/mL) of tryptophan at baseline and following 8 and 24 hours of fermentation per donors 1, 2 & 3 (left to right).

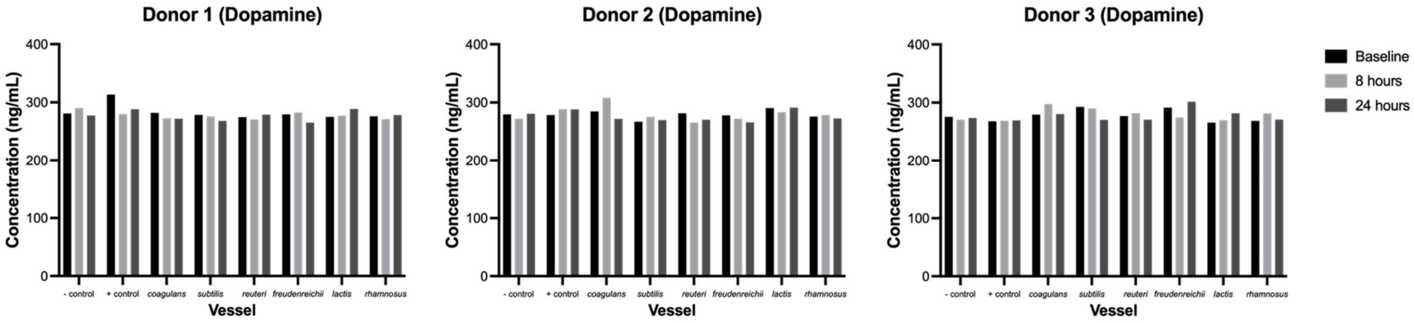

Figure S12. Concentration (ng/mL) of dopamine at baseline and following 8 and 24 hours of fermentation per donors 1, 2 & 3 (left to right).

Table S1. Donor 1, Enumeration of bacteria for by Flow-FISH at baseline (0) and following 8 and 24 hours of fermentation within the negative control, positive control, and six probiotic vessels, represented as log10 cells/mL culture. Target bacteria: Bifidobacterium spp.(BIF), Lactobacillus spp. (LAB), most Bacteroidaceae and Prevotellaceae (BAC), Clostridium coccoides–Eubacterium rectale group (EREC), Roseburia subcluster (RREC), Faecalibacterium prausnitzii (FPRAU), Clostridium cluster IX (PROP), Atopobium-Coriobacterium spp. (ATO), Desulfovibrio (DSV) and Clostridium histolyticum (CHIS).

		**Bacterial groups detected by flow-FISH**										
**Vessel**	**Time (hours)**	**Total bacteria**	**BIF**	**LAB**	**BAC**	**EREC**	**RREC**	**ATO**	**PROP**	**FPRAU**	**DSV**	**CHIS**
Negative control	0	8.13	6.78	6.22	6.69	7.78	7.25	6.01	6.26	7.53	5.96	5.94
	8	7.91	6.74	5.82	6.75	7.46	6.46	6.02	6.27	7.21	3.91	5.49
	24	7.65	6.60	5.91	5.62	7.15	4.85	5.91	5.68	6.15	3.65	5.78
Positive control	0	8.18	6.61	6.40	6.74	7.82	7.39	6.12	6.32	7.58	5.96	5.43
	8	7.80	7.36	5.51	6.08	7.02	6.00	5.57	6.33	7.05	4.80	5.10
	24	7.49	7.01	5.54	5.02	6.28	4.53	5.88	5.75	6.64	4.34	4.10
B. coagulans	0	7.99	6.68	6.09	6.48	7.68	7.15	5.76	5.42	7.37	5.88	4.00
	8	7.78	6.84	5.39	6.27	7.33	6.46	5.47	6.09	7.10	3.78	4.74
	24	7.46	6.73	5.97	5.42	6.90	5.48	5.80	6.21	6.16	3.94	5.36
B. subtilis	0	8.04	6.69	5.93	6.63	7.71	7.14	5.04	6.24	7.44	5.77	5.82
	8	7.87	6.87	5.86	6.29	7.39	6.50	5.93	6.31	7.17	3.87	5.21
	24	7.46	6.73	5.78	5.19	6.84	5.56	5.97	5.86	6.13	3.46	5.76
L. reuteri	0	8.08	6.76	5.56	6.56	7.78	7.28	4.98	6.26	7.44	5.03	5.51
	8	7.79	6.70	5.83	6.67	7.33	6.66	5.67	6.38	7.12	4.75	5.30
	24	7.70	6.67	6.07	6.25	7.23	5.78	5.64	6.22	6.53	4.39	5.82
P. freudenreichii	0	8.06	6.82	6.02	6.17	7.71	6.90	5.24	6.22	7.33	4.67	5.18
	8	6.95	5.02	5.40	4.18	5.33	4.96	3.43	4.21	6.00	3.95	4.13
	24	6.52	4.65	5.14	3.52	4.97	3.98	5.64	5.08	5.19	4.21	4.51
Lc. lactis	0	8.04	6.74	6.23	6.57	7.74	7.12	5.75	6.42	7.44	5.04	6.18

	8	7.46	6.54	5.29	6.56	6.87	5.70	5.33	6.09	6.65	3.46	4.82
	24	7.19	6.44	4.82	5.70	6.13	4.90	5.33	6.21	5.47	3.19	3.89
L. rhamnosus	0	7.94	6.63	6.13	6.58	7.62	7.03	5.98	6.15	7.29	5.78	5.96
	8	7.93	6.62	6.08	6.93	7.57	6.82	4.83	6.10	7.36	4.61	5.27
	24	7.65	5.93	6.06	4.97	7.25	5.79	4.69	6.21	6.71	4.73	5.17

Table S2. Donor 2, Enumeration of bacteria for by Flow-FISH at baseline (0) and following 8 and 24 hours of fermentation within the negative control, positive control, and six probiotic vessels, represented as log10 cells/mL culture. Target bacteria: Bifidobacterium spp.(BIF), Lactobacillus spp. (LAB), most Bacteroidaceae and Prevotellaceae (BAC), Clostridium coccoides–Eubacterium rectale group (EREC), Roseburia subcluster (RREC), Faecalibacterium prausnitzii (FPRAU), Clostridium cluster IX (PROP), Atopobium-Coriobacterium spp. (ATO), Desulfovibrio (DSV) and Clostridium histolyticum (CHIS).

		**Bacterial groups detected by flow-FISH**										
**Vessel**	**Time (hours)**	**Total bacteria**	**BIF**	**LAB**	**BAC**	**EREC**	**RREC**	**ATO**	**PROP**	**FPRAU**	**DSV**	**CHIS**
Negative control	0	7.68	6.88	5.93	6.08	7.00	6.04	5.94	6.20	7.15	5.55	6.24
	8	7.55	6.61	4.75	5.82	6.43	5.25	5.83	6.02	6.89	3.55	5.08
	24	7.37	6.67	4.32	6.06	6.52	4.41	5.70	5.89	6.31	4.57	4.89
Positive control	0	7.63	6.60	4.63	5.85	6.87	5.1	5.44	5.85	7.09	4.41	6.13
	8	7.92	7.52	4.45	5.22	6.52	5.35	7.03	6.32	6.86	4.52	5.68
	24	8.11	8.02	5.11	5.46	6.48	5.66	6.41	6.10	6.28	5.16	5.23
B. coagulans	0	7.81	6.88	5.24	4.89	6.87	5.78	5.38	5.91	7.21	4.41	5.88
	8	7.13	6.42	4.45	5.47	5.63	3.43	6.02	5.87	5.89	3.61	4.28
	24	7.57	7.08	5.01	5.34	5.38	4.27	6.53	6.04	5.75	4.65	4.85
B. subtilis	0	7.67	6.79	4.84	5.20	6.86	5.54	5.30	5.83	7.05	4.27	5.45
	8	7.83	7.00	5.46	6.71	6.40	5.56	6.31	6.40	7.30	5.01	5.79
	24	7.63	6.97	4.88	5.60	6.04	4.63	6.32	6.04	6.77	3.92	4.09
L. reuteri	0	7.98	7.05	5.71	5.64	7.18	6.11	5.88	6.42	7.44	5.58	6.05
	8	7.97	7.18	5.42	6.81	6.81	5.66	6.65	6.57	7.34	4.87	5.68
	24	7.83	7.23	5.23	5.43	6.52	4.83	6.44	6.26	6.75	4.67	5.59
P. freudenreichii	0	7.90	6.89	5.22	5.82	7.11	6.00	5.77	6.19	7.34	4.81	5.18
	8	8.03	7.24	6.38	6.04	6.39	5.38	6.33	6.26	7.07	4.51	4.13
	24	7.98	7.13	6.82	5.38	6.30	4.83	6.61	6.29	6.76	4.68	4.51
Lc. lactis	0	7.93	6.94	5.49	5.53	7.20	5.84	5.51	6.11	7.36	4.53	6.07

	8	7.91	7.13	5.31	6.70	6.71	5.82	6.54	6.54	7.26	4.87	4.82
	24	7.88	7.34	5.11	5.55	6.59	4.48	6.77	6.30	6.83	4.48	3.89
L. rhamnosus	0	7.81	6.83	5.36	5.57	7.06	5.95	5.61	6.15	7.22	4.93	5.90
	8	7.97	7.18	5.42	6.81	6.81	5.66	6.65	6.57	7.34	4.87	5.68
	24	8.04	7.40	5.19	6.17	7.02	5.00	6.78	6.61	6.41	4.89	5.08

Table S3. Donor 3, Enumeration of bacteria for by Flow-FISH at baseline (0) and following 8 and 24 hours of fermentation within the negative control, positive control, and six probiotic vessels, represented as log10 cells/mL culture. Target bacteria: Bifidobacterium spp.(BIF), Lactobacillus spp. (LAB), most Bacteroidaceae and Prevotellaceae (BAC), Clostridium coccoides–Eubacterium rectale group (EREC), Roseburia subcluster (RREC), Faecalibacterium prausnitzii (FPRAU), Clostridium cluster IX (PROP), Atopobium-Coriobacterium spp. (ATO), Desulfovibrio (DSV) and Clostridium histolyticum (CHIS).

		Bacterial groups detected by flow-FISH										
**Vessel**	**Time (hours)**	**Total bacteria**	**BIF**	**LAB**	**BAC**	**EREC**	**RREC**	**ATO**	**PROP**	**FPRAU**	**DSV**	**CHIS**
Negative control	0	7.91	6.52	5.44	5.96	7.48	5.71	5.68	4.82	7.16	4.95	4.91
	8	7.33	5.98	4.54	6.05	6.70	5.06	6.28	4.96	6.77	4.23	4.03
	24	7.75	6.20	5.08	6.60	7.11	5.24	6.63	5.57	7.10	4.06	4.36
Positive control	0	7.98	6.67	5.26	5.78	7.56	6.08	5.75	5.44	7.21	4.46	4.68
	8	8.09	7.66	5.39	6.76	7.20	5.37	7.45	5.54	6.83	4.39	5.45
	24	7.60	6.94	4.20	7.10	6.32	4.07	6.60	5.32	6.14	3.6	3.6
B. coagulans	0	7.60	6.45	4.98	6.07	7.16	5.71	5.51	4.68	6.90	3.60	4.60
	8	7.80	5.00	5.05	6.59	7.31	5.34	6.46	5.20	7.03	3.80	4.75
	24	7.91	6.68	5.19	6.39	7.37	5.24	6.95	6.30	6.71	4.39	4.52
B. subtilis	0	7.81	6.47	5.08	5.71	7.35	5.77	5.65	5.17	7.15	4.85	4.65
	8	7.89	6.46	5.27	6.46	7.46	5.47	6.44	5.82	7.30	4.74	5.61
	24	7.66	6.29	4.77	6.36	7.09	5.09	6.43	5.62	6.77	4.13	4.56
L. reuteri	0	7.93	6.10	5.27	6.24	7.30	6.09	5.58	4.88	7.30	4.71	4.71
	8	7.76	5.87	5.62	6.44	4.24	5.68	5.38	5.06	7.11	4.46	4.76
	24	7.91	5.54	5.77	6.76	7.32	6.08	5.77	6.54	7.26	4.69	3.91
P. freudenreichii	0	7.93	6.21	5.23	6.14	7.38	5.98	5.52	4.63	7.31	5.25	5.18
	8	7.35	6.21	4.55	6.01	5.01	4.81	5.91	4.75	6.72	3.35	4.13
	24	7.49	6.07	5.02	5.79	7.06	3.97	6.32	6.30	6.34	4.09	4.09
Lc. lactis	0	8.07	6.56	5.87	5.87	7.59	6.13	6.00	5.43	7.42	5.02	4.97

	8	7.61	6.35	5.56	6.34	7.13	5.21	6.21	5.48	6.86	4.21	4.69
	24	7.62	6.41	5.10	6.05	6.77	5.28	6.11	5.98	6.48	4.10	3.62
L. rhamnosus	0	7.81	6.32	5.35	5.44	7.32	5.84	5.27	5.22	7.11	4.99	4.71
	8	7.67	6.56	5.59	6.21	6.94	5.16	6.48	5.14	6.85	3.67	4.81
	24	7.53	6.36	5.37	5.94	6.77	4.81	6.02	6.73	6.48	3.83	3.83
